# Is it still necessary to perform measured based pre‐treatment patient‐specific QA for SRS HyperArc treatments?

**DOI:** 10.1002/acm2.14156

**Published:** 2023-10-06

**Authors:** Nina Cavalli, Elisa Bonanno, Giuseppina R. Borzì, Alessia D'Anna, Martina Pace, Giuseppe Stella, Lucia Zirone, Carmelo Marino

**Affiliations:** ^1^ Medical Physics Department Humanitas Istituto Clinico Catanese Misterbianco CT Italy; ^2^ Physics and Astronomy Department E. Majorana University of Catania Catania Italy

**Keywords:** HyperArc, Mobius3D, PSQA, SRS

## Abstract

**Purpose:**

The Mobius3D system was validated as a modern secondary check dosimetry system.

In particular, our objective has been to assess the suitability of the M3D as pre‐treatment patient‐specific Quality Assurance (QA) tool for Stereotactic Radiosurgery (SRS) HyperArc (HA) treatments. We aimed to determine whether Mobius3D could safely replace the measurements‐based patient‐specific QA for this type of treatment.

**Methods:**

30 SRS HA treatment plans for brain were selected. The dose distributions, calculated by Mobius and our routinely used algorithm (AcurosXB v.15.6), were compared using gamma analysis index and DVH parameters based on the patient's CT dataset.

All 30 plans were then delivered across the ionization chamber in a homogeneous phantom and the measured dose was compared with both M3D and TPS calculated one.

The plans were delivered and verified in terms of PSQA using the electronic portal imaging device (EPID) with Portal Dosimetry (PD) and myQA SRS (IBA Dosimetry) detector.

Plans that achieved a global gamma passing rate (GPR) ≥ 97% based on 2%/2 mm criteria, with both Mobius3D and the conventional methods were evaluated acceptable. Finally, we assessed the capability of the M3D system to detect errors related to the position of the Multi‐Leaf Collimator (MLC) in comparison to the analyzed measurement‐based systems.

**Results:**

No relevant differences were observed in the comparison between the dose calculated on the CT‐dataset by M3D and the TPS. Observed discrepancies are imputable to different used algorithms, but no discrepancies related to goodness of plans have been found.

Average differences between calculated (M3D and TPS) vs measured dose with ionization chamber were 2.5% (from 0.41% to 3.2%) and 1.81% (from 0.66% to 2.65%), for M3D and TPS, respectively.

All plans passed with a gamma passing rate > 97% using conventional PSQA methods with a gamma criterion of 2% dose difference and 2 mm distance‐to‐agreement. The average gamma passing rate for the M3D system was determined to be 99.4% (from 97.3% to 100%). Results from this study also demonstrated Mobius has better error detectability than conventional measurement‐based systems.

**Conclusion:**

Our study shows Mobius3D could be a suitable alternative to conventional measured based QA methods for SRS HyperArc treatments.

## 1 INTRODUCTION

Patient‐specific QAs are important steps of the radiation therapy process to identify discrepancies between calculated and delivered treatment plans.^1^

Since the introduction of Intensity‐Modulated Radiation Therapy and/or Volumetric‐Modulated Arc Therapy (IMRT/VMAT) techniques, various procedures for patient‐specific QA based on measurement and calculation methods have been proposed,^2^ including independent monitor unit (MU) calculations for IMRT/VMAT.^3^

The goal of patient‐specific QA for IMRT/VMAT plans is to verify the accuracy of dose calculation and to detect clinically relevant errors during the delivery of radiation doses, thereby ensuring the safety of patients. Several papers have been published during last years regarding IMRT/VMAT clinical implementation and many professional organizations^4^, ^5^, ^6^, ^7^ have strongly recommended patient‐specific IMRT/VMAT QA be employed as part of the clinical IMRT process. In particular, in a comprehensive white paper published in 2011, the importance of conducting pre‐treatment validation for patient safety was emphasized. However, the paper did not explicitly outline the specific methods for conducting such validation.^6^ Different methods besides measurements have been proposed, including independent computer calculations, check‐sum approaches, and log file analysis^3^, ^8^, ^9^, ^10^, ^11^, ^12^, ^13^

In a Report of the AAPM task group 120, Low et al. reports strengths and weaknesses of different dosimetric techniques, regarding data acquisition for commissioning patient‐specific measurements.^14^ The value of patient‐specific IMRT/VMAT QA has been debated among physicists^8^, ^12^, ^15^, ^16^ especially whether computational methods can replace physical measurements. In a “point/counterpoint” debate, Siochi discusses^16^ the possibility to perform the patient specific portion of “IMRT QA” using software only. In fact, he underlines the potentiality of an approach that treats each subsystem of Radiation Therapy process separately. If QAs are performed on the delivery system at a high enough frequency to ensure that the system is operating as needed to achieve the accuracy required for IMRT/VMAT, verification on the patient's planned dose distributions can be performed using an independent, secondary, composite dose calculation system. According to this approach, quality control is also performed on the patient's treatment delivery parameters in the delivery system's database, to ensure that they match the values in the treatment plan.^16^, ^17^

Mobius3D (M3D) is the Varian (Varian Medical Systems Inc., Palo Alto, California, USA) solution of a secondary independent and log file based dose verification system. It performs a full recalculation of dose on the patient CT and allows for quality assurance of the treatment plan by offering a “delivered dose” calculation, generated using the M3D model and treatment machine's log files.^18^, ^19^

In a recent study, Hasse et al.^20^ investigated the possibility to use M3D dose calculation software to reduce the number of physical measurements and the required amount of on‐site personnel, during corona virus disease, while maintaining patient safety.

The study demonstrates that using M3D with appropriate threshold dose can substantially reduce the number of plans that needs measured based patient specific QA (27.4% for a TrueBeam over a 212 treatments plans analyzed).

Basavatia et al.^21^ studied the possibility to use M3D/MFX as not only a pretreatment secondary check but as an alternative to measurements‐based patient‐specific QA for IMRT/VMAT. This study concerns standard fractionated and SBRT (lung and spine) radiation treatments and compares M3D results against EPID and Radiochromic film measured based results. Authors conclude that M3D could be a suitable alternative to conventional QA methods when using the 3%/3 mm gamma criterion, according to the different features of the detectors used for comparison.

M3D was validated for a variety of radiation therapy techniques, IMRT/VMAT/TomoTherapy, demonstrating to be a safely pre‐treatment calculation‐based verification system.^19^, ^20^, ^21^, ^22^, ^23^, ^24^

However, there is a lack of investigation regarding the appropriateness of M3D as patient‐specific pre‐treatment verification system for stereotactic brain radiation treatment with a high dose in a single fraction.

According to these considerations, we evaluate the appropriateness of M3D/MFX as pre‐treatment patient specific QA for HyperArc (HA) treatments, establishing if Mobius3D can safely replace the measurement‐based patient‐specific QA for this kind of treatments.

HyperArc is the recent solution provided by Varian Medical System for SRS dose delivery.^25^, ^26^, ^27^ This new tool incorporates several specialized functions for generating a HyperArc VMAT (HA‐VMAT) plan with a minimal workload including automated settings for the location of the isocenter, non‐coplanar beam arrangement, collimator angles, and the optimization process. The use of HA affords the possibility of delivering a more conformal dose to the target while reducing doses to surrounding tissues as far as possible.

The purpose of this study is to assess the validity of the M3D as an alternative to the standard measurements‐based approach limited to the brain stereotactic HA treatments.

## 2 MATERIALS AND METHODS

Thirty SRS HA‐VMAT plans for brain were selected . All studied treatments plans were achieved with no‐coplanar arcs, using a 6 MV‐ FFF (Flattening Filter Free) photon beam and a dose rate of 1400 MU/Gy, provided by a TrueBeam 2.7 (Varian Medical System) with a High Definition 120‐Leaf Multi Leaf Collimator (HD120 MLC).

Plans were designed and optimized with VMAT technique using the HA module in the Eclipse TPS (Varian Medical System, Palo Alto, California, USA) and calculated with the configured algorithm Acuros XB (AXB*) version 15.06.06* and a calculation grid size of 1.25 mm. As all planes were single targets, the isocenter was positioned within the target itself. The mean target volume of interest was 1.5 cc (min. 0.10 cc; max. 7.30 cc), while the mean MU/Gy value was 270.9 (min. 226.4; max. 372.6). The prescription dose was 20−22 Gy as a single fraction.

All plans were delivered and verified with conventional measurements‐based approach: ionization chamber CC04 (IBA, Dosimetry, Germany) with a 0.04 cm^3^ volume, EPID with Portal Dosimetry (Varian Medical Systems Inc., Palo Alto, California, USA), and myQA SRS digital detector array with Patient QA software package (IBA, Dosimetry, Germany).^28^

The appropriateness of M3D as an alternative to measurement‐based patient specific QA, was evaluated considering verified plans that passing 3D global gamma analysis with Mobius3D and the conventional presented methods using a global gamma passing rate (GPR) ≥97% and 2%−2 mm criteria.

### 2.1 Mobius3D analyses

M3D has been designed to perform a full check of the ability of the TPS to accurately account for patient heterogeneities. In fact, M3D acquires patient CT and plan details from primary TPS and then it performs a 3D dose calculation on the patient CT‐dataset, using a collapsed cone convolution superposition (CCCS) algorithm and an independent beam model.

M3D compares its dose calculation results to the TPS calculated dose, in the *Plan Check Module*, providing an independent evaluation on the accuracy of the TPS dose calculation algorithm.

With the purpose to establish the strength of our calculation‐based pretreatment patient QA methods for HA, we also evaluated results of Mobius FX (MFX), in the *QA Check Module*.

MFX uses the same algorithm as M3D, but retrieves the trajectory log files from the linear accelerator to recalculate the dose on the patient's CT, allowing to check possible discrepancies between calculated dose (M3D/TPS) and delivered dose.

The trajectory log files include jaw, MLC, couch, and gantry position as well as the MUs, that are recorded during plan delivery.

M3D analysis was performed in two different steps:

In the first step plans calculated with Eclipse TPS were exported to Mobius3D System to proceed with the evaluation of *Plan Check Module*.

For all selected HA plans, dose distributions calculated on the patient's CT‐ dataset by M3D (*CCCS* algorithm) and TPS (*AXB v.15.6* algorithm) were compared.

The dose distributions calculated by M3D and TPS were evaluated using the following metrics provided by the *Plan Check* module: target D_Mean_ percent difference, target D_90%_ percent difference, and 3D global gamma passing rate over the entire dataset. For 3D gamma evaluation the used gamma criterion was 2%−2 mm, with a threshold dose of 10%.

In a second step, for all treatments, verification plans were created and calculated into the Mobius Verification Phantom (MVP), a homogeneous phantom containing ionization chamber inserts.

**FIGURE 1:**
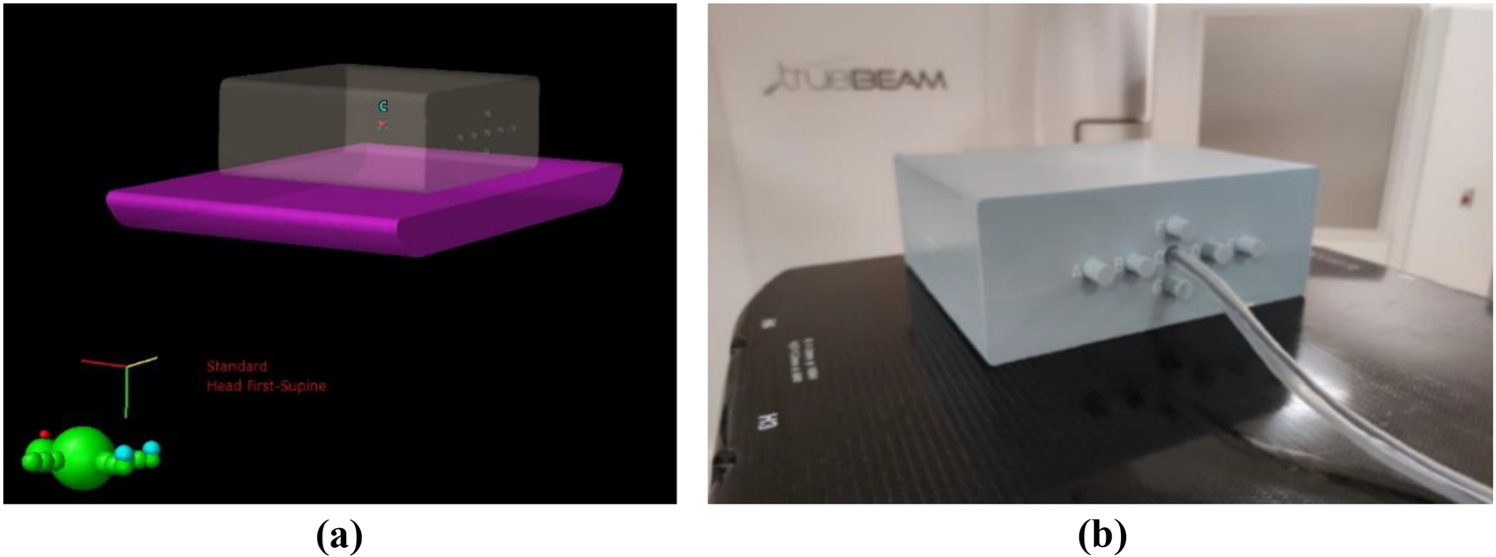
(a) ROI C. (b) Ionization chamber in the MVP Phantom.

In the *Plan Check Module*, TPS calculated dose on the MVP was compared to M3D calculated one. The evaluation was performed in a ROI (Region of Interest, structure *C* in Figure 1a) corresponding to the MVP's insert, in which the chamber was located (Figure 1b). For each plan, the isocenter was positioned so that the center of the target corresponded to the center of the chamber's active volume.

All plans were then delivered across the ionization chamber located in point C of the MVP and percentage dose differences between TPS/M3D calculated dose and chamber measured one were quantified.

### 2.2 EPID based portal imaging analysis

The electronic portal imaging device (EPID‐Varian Medical System aS1200 model) was used for all plans acquisition and the analysis was performed using the VARIAN Portal Dosimetry (PD) *v. 2.22.5.0* software application. The a‐Si1200 EPID detector has an active area of 40 × 40 cm^2^ with 1190 × 1190 pixels array and a pixel spacing of 0.336 mm.

Predicted dose distributions calculated by TPS were compared to those acquired by EPID and analyzed with PD. A global gamma evaluation was performed using a 2%−2 mm criterion with a 10% threshold dose. The gamma analysis was performed on each treatment arc and a mean value on the number of the arcs was reported for each plan. Accettable results were evaluated considering a gamma passing rate ≥ 97 %.

### 2.3 *myQA* SRS detector

**FIGURE 2:**
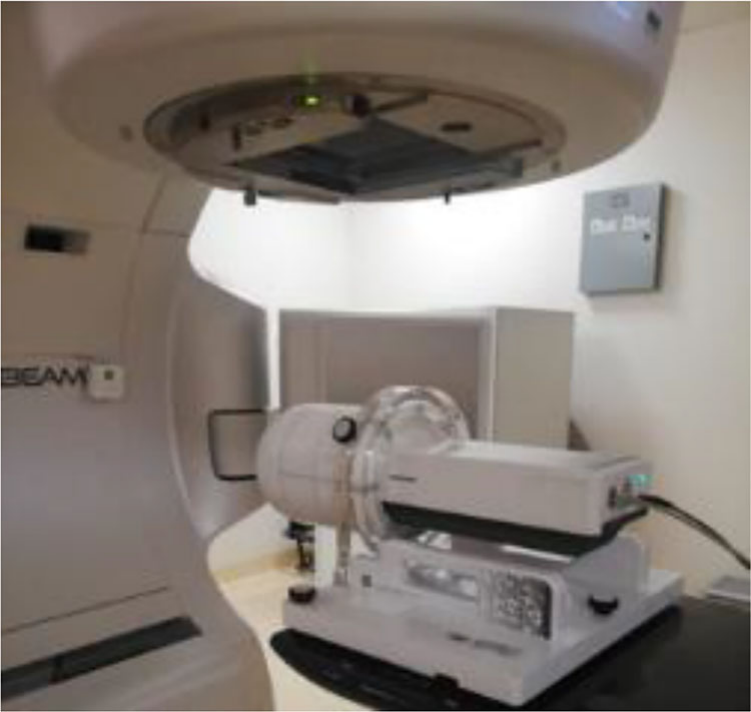
myQA SRS high‐resolution detector (IBA dosimetry).

*myQA* SRS is a measurement detector for PSQA of SRS/SBRT treatments. The measurement sensors of *myQA* SRS are monolithic solid‐state semiconductor arranged in a 2D array and the detector is optimized to measure 2D dose maps of small fields with high resolution. The sensor layout is a grid of 300 × 350 pixels with a spacing of 400 μm in an area of 120 mm x 140 mm (active area). The *myQA* SRS is located inside the *myQA* SRS phantom (Figure 2): this provides a medium of homogenous build‐up and backscatter material.

A verification plan was created and calculated in the myQA SRS phantom, for each HA plan, using *AXB* and the *“field based”* setting.

Each plan was delivered on the myQA SRS phantom, taking also into account the couch rotation. A calibration output factor was performed before of each measurement session, to taking into account the daily output Linac variation.

2D measured dose distribution at the isocenter plane, for each arc, was compared with the calculated field dose exported on the *myQA platform* from TPS. A global gamma analysis index was performed using 2%−2 mm criteria, with 10% threshold dose.

### 2.4 MobiusFX

To test the robustness of the M3D System as pretreatment QA alternative to the conventional measurement‐based method, all plans were delivered and analyzed also using the MFX module.

During the first delivery fraction of plans, the Mobius FX in the *QA Check* module, compares the delivered 3D dose to the 3D TPS and M3D calculated dose. These results are calculated using the M3D dose calculation algorithm and the positional measurements contained in treatment log files.^18^ The treatment log files check is also performed by MFX during all subsequent treatment fractions, in case of more fractions.

The Mobius FX software determines root mean square (RMS) errors by comparing “Delivered” values to “Set” values.

The RMS error evaluation helps identify how well the machine has delivered the treatment plan as defined by the machine as interpreted from the RT plan DICOM file; it condenses a set of errors into a single representative value. MFX calculates RMS errors for individual leaves in files, ranges of gantry angles in files, and entire leaf banks in a collection of files.

In this phase of our study, the MFX module was used as guide to identify differences due to calculation step from the differences regarding linear accelerator performances: if the delivered doses evaluated by MFX exactly correspond to the M3D doses, it means that (according to Mobius features) the accelerator has accurately delivered the prescribed treatment plan.

Evaluation of MFX delivered dose was performed in terms of target D_90%_ percent differences and target D_mean_ percent differences between MFX delivered and TPS calculated dose.

Furthermore, the DVH tool checks the TPS, M3D and MFX dose values in the organ at risk (OAR) against limits established in AAPM task group (TG‐101) protocols.^29^

### 2.5 Sensitivity to intentional errors in HyperArc plans

To ensure a comprehensive approach, we investigated the differences in sensitivity between Mobius3D and the two measurements‐based systems with the purpose to find detection thresholds for both approaches. We checked the ability of the studied systems to detect intentional errors related to the high definition (HD) MLC position by inducing errors of various magnitudes.

Ten of the 30 total studied plans were selected and new plans with intentional errors of different sizes regarding leaf positions were generated, using an in‐house binary plugin script implemented using the Eclipse scripting application programming interface (ESAPI).

For each plan, MLC positioning errors were introduced, including widening of entire leaf bank A of 0.5 , 0.8 , and 1 mm, for a total of 30 investigated plans.

The impact of the errors in the modified plans was assessed using gamma analysis with local 2%/2 mm and local 2%/1 mm (with 10% threshold dose) criteria, for both the measurement‐based methods.

To determine systems’ ability to detect introduced errors, we evaluated the gamma passing rate between the original and modified measured dose distribution for each plan.

To evaluate the M3D's sensitivity to detect introduced errors we analyzed results of MFX, in the QA Check Module, for all modified plans. The study of results with M3D system concerned the “*Delivery Beam Information*” contained in the QA Check module. Among other things, this module contains information on the discrepancy between planned and delivered MLC positions, with an alert level of 0.4 mm for SBRT/SRS protocol. Moreover, the 3D global gamma analysis passing rate (2%−2 mm criteria), between correct calculated plans and the intentional modified delivered ones and how much it degraded as the error increased, was evaluated by M3D.

### 2.6 PSQA using PD/Epid and M3D: time‐consuming

A time‐consuming evaluation was performed to quantify differences between PSQA process using Portal Dosimetry with Epid (starting from the creation of the plan verification up to GPR analysis) and M3D (starting from sending plan to M3D up to GPR analysis).

## 3 RESULTS

### 3.1 Mobius3D analysis

Results regarding dose distributions calculated on the patient's CT‐ dataset by Mobius3D and our TPS were evaluated in terms of target D_mean_ and target D_90%_.

For target D_Mean %_ differences range from a minimum of 0.01% to a maximum of 2.96%, with an average value of 1.95%; for target D_90%_ differences range from 0.13% to 2.91%, with an average value of 2.06%.

Observed percentage differences regarding the comparison between calculated dose on the CT‐dataset by M3D and TPS are imputable to different used algorithms, but no discrepancy related to goodness of plans has been found. The 3D global gamma passing rate, using a 2%−2 mm criteria, over the entire dataset was evaluated: the average passing rate over all the 30 HA plans was 99.41%, from a minimum of 97.3% to a maximum, of 100.0%. Table 1 reports absolute % differences between calculated (M3D and TPS) and measured dose with ionization chamber in the MVP, in terms of minimum, maximum, and average percent differences over 30 plans.

**TABLE 1:** Dose differences between M3D and TPS calculated dose values and ionization chamber measured ones; MFX expected dose versus chamber measured dose.

	M3D vs. chamber %Δ	TPS vs. chamber %Δ
Average	2.52%	1.81%
Minimum	0.41%	0.66%
Maximum	3.2%	2.65%

### 3.2 EPID‐based portal imaging and *myQA* SRS detector analysis

Table 2 reports global gamma analysis evaluation performed across the two measurement‐based conventional methods. Gamma analysis was performed for each arc and the average value of the gamma passing rate is reported for each method. The gamma criterion was established at 2%−2 mm for both the used measurement‐based methods. All plans passed the gamma evaluation with conventional methods, showing a mean gamma passing rate of 99.5% and 99.3%, for EPID and *myQA* SRS, respectively, from a minimum of 97.8% to a maximum of 100% for EPID and from a minimum of 97.7% to a maximum of 100% for *myQA* SRS. For comparison, Table 2 also reports the results related to the M3D global gamma evaluation mentioned in **3.1**. All verified plans satisfy the gamma analysis passing rate ≥ 97% with Mobius3D and with both used conventional measurement‐based methods.

**TABLE 2:** Average, minimum, and maximum GPR performed with EPID, myQA SRS detector, and Mobius3D System.

	EPID‐PD	myQA SRS	M3D
Average GPR	99.5%	99.3%	99.4%
Minimum GPR	97.8%	97.7%	97.3%
Maximum GPR	100.0%	100.0%	100.0%

**FIGURE 3:**
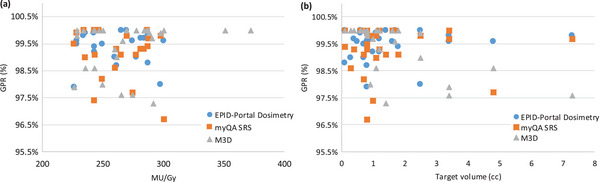
(a) %GPR versus MU/Gy. (b) %GPR versus target volume (cc).

Figure 3a,b shows how %GPR results are independent of MU/Gy and target volume.

### 3.3 MobiusFX

Table 3 reports differences between TPS calculated dose and MFX delivered dose detected during the first delivery fraction of each HA plan, in the *QA Check* module. Results are reported in terms of the maximum, minimum, and average value of discrepancies over the 30 HA plans.

**TABLE 3:** Differences between TPS and delivered dose in terms of target D_Mean_ and target D_90%_.

	TPS vs. MFX (*Delivered*) %Δ	
	Target D_Mean_	Target D_90%_
Average	0.52%	‐0.72%
Minimum	0.14%	0.16%
Maximum	3.10%	3.04%

The observed differences between TPS and delivered dose are ≤ 3.1% for all plans for both target D_Mean_ and target D_90%._

The MFX analysis, through TrueBeam's log files, did not highlight discrepancy for all 30 HA plans. This allows us to verify, in this first approach to a calculation‐based pre‐treatment patient‐specific QA, that the accelerator has accurately delivered the prescribed treatment plan. All analyzed OARs through DVH tool respect the AAPM TG‐101 limits for TPS, M3D, and MFX evaluated dose.

### 3.4 Sensitivity to intentional errors in HyperArc plans

Table 4 reports results regarding plans with MLC's positioning errors. Of the 30 modified plans, EPID system identified all failed plans with the 1 mm MLC's error position, four failed plans with 0.8 mm error and none of the plans with 0.5 mm error, using a gamma criterion of 2%−1 mm (10% threshold dose). For plans with 1 mm error the mean gamma passing rate was 89.10 % from a minimum of 78.83% to a maximum of 96.89%. For plans with 0.8 mm error the mean gamma passing rate was 96.17% from a minimum of 89.13% to a maximum of 99.73%. Finally, for plans concerning 0.5 mm error position, the mean gamma passing rate evaluated with EPID was 99.33% from a minimum of 97.23% to a maximum of 100%. Regarding the analysis with *myQA* system and 2%−1 mm criteria (10% threshold dose), the 1 mm MLC error position was identified for all plans with a mean gamma passing rate of 82.31 % [68.7–95.1%]; the 0.8 mm error was identified for 7 of 10 plans with a mean gamma passing rate of 90.34 % [72–97.65%]; the 0.5 mm error was identified for three plans with mean gamma passing rate of 96.56 % [82.2–99.5%].

**TABLE 4:** Local Gamma passing rate for measurements‐based systems for plans with errors.

EPID‐Portal Dosimetry (Local Gamma 2%−1 mm)			
	Cor. vs.1 mm Error	Cor. vs. 0.8 mm Error	Cor. vs. 0.5 mm Error
Plan I	**95.88%**	97.55%	99.45%
Plan II	**83.32%**	**92.35%**	99.30%
Plan III	**89.50%**	**96.50%**	99.53%
Plan IV	**79.73%**	**89.13%**	97.23%
Plan V	**96.63%**	98.83%	99.73%
Plan VI	**92.15%**	97.23%	99.55%
Plan VII	**78.83%**	98.78%	100.0%
Plan VIII	**96.89%**	99.73%	100.0 %
Plan IX	**96.05%**	98.90%	99.95 %
Plan X	**81.97%**	**92.70%**	98.60%

myQA‐SRS (Local Gamma 2%−1 mm)			
	Cor. vs. 1 mm Error	Cor. vs. 0.8 mm Error	Cor. vs. 0.5 mm Error
Plan I	**89.90%**	97.65%	98.40%
Plan II	**68.70%**	**82.20%**	98.20%
Plan III	**82.70%**	**93.80%**	99.30%
Plan IV	**83.70%**	**92.30%**	99.20%
Plan V	**84.40%**	97.60%	99.00%
Plan VI	**95.10%**	97.65%	99.50%
Plan VII	**88.30%**	**94.90%**	99.10%
Plan VIII	**72.20%**	**72.00%**	**82.20%**
Plan IX	**71.30%**	**81.70%**	**95.00%**
Plan X	**86.80%**	**93.60%**	**95.70%**

Values in bold < 97%.

The local gamma analysis with 2%−2 mm (10% threshold dose) shows a lower sensitivity for both the measured based systems: of the 30 plans with introduced leaf bank errors, *myQA* and EPID systems detected four plans regarding 1 mm error and none with 0.5 mm; for 0.8 mm error *myQA* detected 2/10 failed plans while only one plan with error was detected by EPID. Table 5 reports results regarding “*Beam Information*” provided by MobiusFX for plans delivered with intentional errors. Results are expressed in terms of MLC value that exceeds the alert level set to 0.4 mm, for each plan.

**TABLE 5:** MLC value that exceeds alert level.

MFX The MLC value always exceeds the alert level			
	1 mm Error	0.8 mm Error	0.5 mm Error
Plan I	**1.16 mm**	**0.97 mm**	**0.80 mm**
Plan II	**1.12 mm**	**0.93 mm**	**0.63 mm**
Plan III	**1.32 mm**	**1.12 mm**	**0.82 mm**
Plan IV	**1.14 mm**	**0.93 mm**	**0.63 mm**
Plan V	**1.32 mm**	**1.12 mm**	**0.82 mm**
Plan VI	**1.24 mm**	**1.04 mm**	**0.74 mm**
Plan VII	**1.24 mm**	**1.05 mm**	**0.75 mm**
Plan VIII	**1.14 mm**	**0.93 mm**	**0.63 mm**
Plan IX	**1.14 mm**	**0.94 mm**	**0.64 mm**
Plan X	**1.10 mm**	**0.89 mm**	**0.59 mm**

For 3D global gamma analysis performed by MFX, the mean gamma passing rate was 83.30% [73.50%−89.0%] for plans with 0.5 mm MLC positioning error, 70.63% [62.90%−73.90%] for 0.8 mm error, and 60.71% [52.9%−67.5%] for plans with 1 mm error.

### 3.5 PSQA using PD/Epid and M3D: Time‐consuming

The necessary time to create, deliver and analyze verification HA plans using the conventional Portal Dosimetry and Epid PSQA approach is 10.5 ± 0.6 min (it depends on calculated MU/dose prescription), while the necessary time to send and to evaluate HA plans using M3D approach is 2 min.

## 4 DISCUSSION

M3D performs a complete and independent evaluation on the accuracy of the primary TPS's algorithm,^21^ carefully evaluating the dose distribution for all types of radiotherapy treatment plans. The software provides helpful tools for plan evaluation and dose calculation verification including the DVH tool, gamma analysis, and region of interest statistics.

The gamma analysis tool performs 3D gamma analysis between the TPS and M3D dose calculation. It has a slice slider that allows the user to move one plane at a time through the 3D gamma analysis and determine areas of disagreement between the TPS and M3D.

Furthermore, the region of interest statistics provides the user with the gamma passing rates within each of the contoured structures as well as the mean dose in each structure. These tools can be very helpful, verifying that the dose calculation is accurate, and identifying regions where the TPS calculation may be inaccurate.

For purpose of this study, it is very important to understand if potential calculated dose differences found, on the patient CT‐dataset, between TPS and M3D, are imputable to different used algorithms, or the discrepancies are related to goodness of plans.

The CCC dose engine determines dose deposition by a three‐dimensional convolution/superposition of the Total Energy Released per unit Mass (TERMA) with a dose spread function. The TERMA is determined by projection of the beam energy fluence through the patient CT volume. The effects of changes in tissue composition on dose distribution are approximated by scaling the dose spread function by the radiological distance to account for the presence of heterogeneities with respect to scattered radiation. The heterogeneity corrections are approximate and have been shown to underestimate (or overestimate) dose at bone/air/tissue interfaces.^30^, ^31^, ^32^

Acuros XB, similar to the Monte Carlo algorithm (often accepted as the golden standard), explicitly models the physical interaction of radiation in media and solves the Linear Boltzmann Transportation Equations (LBTE) to calculate the energy‐dependent fluence.^33^, ^34^

Several authors report the impact of Acuros XB on IMRT/VMAT stereotactic radiotherapy and show that AXB improves the dose calculation accuracy from 41% to 6% at the air/tissue interface^35^ when compared to other algorithms. All of the studies show the advantage of Acuros XB compared with other algorithms, different form Monte Carlo, in terms of accuracy.^36^

In the case of HA treatments, a level of automation of the TPS also covers planning through the use of SRS normal tissue objective (SRS NTO). The SRS‐NTO is designed to control dose fall‐off and dose bridging at the level of 17% of the prescription dose. The AutoNTO uses a cost function that defines the shape of the dose fall‐off, which is controlled by a set of internal parameters that are dynamically adapted during optimization.

Small field dosimetry introduces some issues such as partial occlusion of the primary source and loss of Charged Particle Equilibrium (CPE) on the central axis, and detector related, relative to its dimensions with respect to the field and its perturbation effects on the particles spectra.^37^, ^38^ These conditions, resulting in overlapping penumbrae over the detector volume, may affects its readings, thus the accuracy of the treatment planning system (TPS) in predicting dose distributions. Dosimetric inaccuracies may lead to poor outcomes for patients.

Based on this consideration, our work aims to establish the strength of pre‐treatment calculation‐based patient specific QA, using M3D and MFX systems, for HA brain radiation treatments. Furthermore, considering that MFX uses the same M3D algorithm to recalculate delivered plans, it is possible to assume M3D as primary PSQA system without the necessity to run MFX prior to the first treatment as part of the PSQA process; obviously this could be possible only with a severe quality control program on MLC and generally on TrueBeam systems and/or providing periodical PSQA verifications intended from the reproducibility point of view (i.e., reference treatment plans delivered periodically).

The brain is an anatomical region without critical air/tissue interface such as the lung region, for example, in which algorithms describe dose transport in a deeply different way.

The most critical situation (in terms of “*algorithm's behavior*”) regarding an HA treatment is that lesion could be located in the bone/tissue interface.

However, the bone‐tissue interface is less critical in terms of dose calculation accuracy for the algorithms^39^ with respect to the air‐tissue interface, for which it could be difficult to understand if calculated TPS/M3D differences can be attributable to the different nature of algorithms or to realistic discrepancies that make the plan not clinically acceptable.

In this work, comparison in MVP phantom between ionization chamber measured dose and TPS/M3D calculated dose shows good agreement for all studied HA plans with discrepancies ≤ 3.2%. From this point of view, our method is very similar to an E2E approach, and our results satisfy tolerances recommended by AAPM's Practice Guideline 9.a.^40^

Considering the high resolution of the two detector systems (EPID and myQA SRS) used, we are confident that our plans evaluation using M3D for HA treatments can be safely expressed.

It is evident that an exact comparison is not possible, since each of the used measured based methods has a different QA setup and different intrinsic features. For the same reason, the used gamma analysis is not the same for all the studied approaches (3D global for M3D, 2D global for *myQA* SRS and EPID), but each system was used according to the commissioning process followed for their routinely employment in our institute.

The *AAPM TG No. 218* ^41^ recommends a gamma passing rate ≥95% with 3%/2 mm and 10% dose threshold, using global normalization, as universal tolerance limits for IMRT measurement‐based verification QA, suggesting tighter tolerances for SRS/SBRT cases. Taking into account these recommendations and results reported in literature,^41^, ^42^, ^43^, ^44^ an universal tolerance limit of 2%−2 mm was applied in this study for all used methods and a gamma passing rate ≥97% was considered accettable.

All the used patient specific QA methods in this study have the same end‐goal, that is, to evaluate whether a treatment plan is clinically deliverable and the overall pass/fail results are in good agreement in our analysis. In fact, all studied plans showed a gamma passing rate > 97% with M3D, using a 2%−2 mm criterion. The same gamma passing rate results have been found using the conventional measured based pretreatment patient specific QA methods. Results given from M3D and from the two detector systems are consistent, or rather the gamma passing rate found for each plan by the detectors and by M3D, shows the same trend.

Furthermore, as additional system's accuracy check, an evaluation on MFX information has been performed. For all 30 HA plans, delivered dose and its distribution evaluated by MFX was compared to the TPS calculated ones. The verification process, using the M3D dose calculation algorithm and position measurements from the treatment log file, confirms that the treatment accelerator has accurately delivered the prescribed treatment plan.

No discrepancy has been observed and the goodness of our calculation‐based pre‐treatment patient‐specific QA is therefore confirmed by the treatment log file check performed by MFX.

To complete our study on the strength of M3D, we performed an accurate analysis on the ability of Mobius3D system to detect delivery intentional errors on the MLC leaf bank positioning, compared to the other two measurement‐based systems.

Au et al.^45^ investigated the Mobius system's ability to detect intentional errors for MLC bank off‐set in the range of 1–10 mm. However, SRS brain targets are very small and the related field size can be < 1 × 1 cm^2^. Therefore, for this kind of treatments, it becomes fundamental testing the M3D system's capability to detect errors ≤ 1 mm.

In this work, M3D\MFX system, has detected the position of MLC exceeding the set value from calculation for all 30 analyzed plans with intentional errors. Moreover, the 3D global gamma analysis performed by M3D\MFX showed a decreasing passing rate as the error magnitude increased. Results from this study suggest that 2%/2 mm global gamma analysis has sufficient sensitivity to detect introduced errors in MLC positioning for SRS HyperArc treatments.

This work demonstrates that for HA treatment plans the M3D\MFX system could be a “solid” alternative to conventional measurement‐based pre‐treatment patient‐specific QA.

Obviously, all the steps related to the validation and periodic quality control program of the accelerator (particularly careful to the small fields dosimetry) with appropriate frequency,^40^ cannot be replaced or avoided to guarantee the safety and efficacy of the SRS/SBRT treatments. Moreover, to assure the multiple means of detection for safety, a set of HA‐VMAT treatment plans could be weekly delivered and analyzed to assure the radiation treatment's reproducibility accuracy.

## 5 CONCLUSION

Pre‐treatment patient specific QA using the Mobius3D system could be safely implemented in our clinical practice, for HyperArc treatment plans.

The Mobius3D's tools are very useful and they allow to perform a complete check of the plan starting from the calculation step up to the accelerator's delivery treatment.

The M3D\MFX calculation‐based method has demonstrated to be accurate and very time sparing, allowing to evaluate the opportunity to check all the planned patients with HA in our institute, reducing machine time spent for measurement. This represent a very important additional advantage in a department with a considerable workload.

## AUTHOR CONTRIBUTIONS

Conception and design: Nina Cavalli, Carmelo Marino. Data collection: Nina Cavalli, Giuseppina Rita Borzì, Giuseppe Stella, Lucia Zirone, Martina Pace. Data analysis and interpretation: Nina Cavalli, Elisa Bonanno, Carmelo Marino, Giuseppe Stella. Manuscript writing: Nina Cavalli, Giuseppe Stella, Carmelo Marino. Final approval of manuscript: Carmelo Marino

## CONFLICT OF INTEREST STATEMENT

All the authors declare no conflict of interest or financial interests.
